# Interictal Epileptiform Discharges are Task Dependent and are Associated with Lasting Electrocorticographic Changes

**DOI:** 10.1093/texcom/tgab019

**Published:** 2021-03-20

**Authors:** Stephen Meisenhelter, Robert J Quon, Sarah A Steimel, Markus E Testorf, Edward J Camp, Payam Moein, George W Culler, Robert E Gross, Bradley C Lega, Michael R Sperling, Michael J Kahana, Barbara C Jobst

**Affiliations:** Department of Neurology, Dartmouth-Hitchcock Medical Center, Lebanon, NH 03766, USA; Department of Neurology, Geisel School of Medicine at Dartmouth College Hanover, NH 03755, United States; Department of Neurology, Geisel School of Medicine at Dartmouth College Hanover, NH 03755, United States; Department of Neurology, Geisel School of Medicine at Dartmouth College Hanover, NH 03755, United States; Thayer School of Engineering at Dartmouth College, Hanover, NH 03755, United States; Department of Neurology, Dartmouth-Hitchcock Medical Center, Lebanon, NH 03766, USA; Department of Neurology, Dartmouth-Hitchcock Medical Center, Lebanon, NH 03766, USA; Department of Neurology, Dartmouth-Hitchcock Medical Center, Lebanon, NH 03766, USA; Department of Neurosurgery, Emory University, Atlanta, GA 30322, United States; Department of Neurosurgery, University of Texas-Southwestern, Dallas, TX 75390, United States; Department of Neurology, Thomas Jefferson University, Philadelphia, PA 19144, United States; Department of Psychology, University of Pennsylvania, Philadelphia, PA 19104, United States; Department of Neurology, Dartmouth-Hitchcock Medical Center, Lebanon, NH 03766, USA; Department of Neurology, Geisel School of Medicine at Dartmouth College Hanover, NH 03755, United States

**Keywords:** attention, cognition, epilepsy, interictal epileptiform discharge, memory

## Abstract

The factors that control the occurrence of interictal epileptiform discharges (IEDs) are not well understood. We suspected that this phenomenon reflects an attention-dependent suppression of interictal epileptiform activity. We hypothesized that IEDs would occur less frequently when a subject viewed a task-relevant stimulus compared with viewing a blank screen. Furthermore, IEDs have been shown to impair memory when they occur in certain regions during the encoding or recall phases of a memory task. Although these discharges have a short duration, their impact on memory suggests that they have longer lasting electrophysiological effects. We found that IEDs were associated with an increase in low-frequency power and a change in the balance between low- and high-frequency oscillations for several seconds. We found that the occurrence of IEDs is modified by whether a subject is attending to a word displayed on screen or is observing a blank screen. In addition, we found that discharges in brain regions in every lobe impair memory. These findings elucidate the relationship between IEDs and memory impairment and reveal the task dependence of the occurrence of IEDs.

## Introduction

Interictal epileptiform discharges (IEDs) correlate with transient cognitive impairment in epilepsy (Binnie and Marston 1992; Landi et al. 2019). Studies have found that when IEDs occurred in lateral temporal and parietal regions during the encoding and recall phases of a free recall task, subjects had momentarily impaired memory performance (Krauss et al. 1997; Horak et al. 2017; Ung et al. 2017). There is further evidence that IEDs cause impairment only when they occur contralateral to the seizure onset zone (Rausch et al. 1978; Kleen et al. 2013), suggesting that the interruption of memory is due to acute impairment of functional tissue. In this study, we investigated the occurrence of IEDs and the conditions under which they cause memory impairment.

The factors that control the timing and occurrence of IEDs remain unclear, but there is some evidence that attention can modulate the occurrence of IEDs. During a study to assess the impact of IEDs on driving ability, experimenters noted that the subjects had fewer IEDs during the driving task than when sitting idle at stop signs (Kasteleijn-Nolst Trenité et al. 1987). Furthermore, there is evidence of a relationship between epilepsy and attention disorders (Socanski et al. 2013), and IEDs and transient cognitive impairment (Aldenkamp 1997; Kleen et al. 2013), suggesting that there may be a bidirectional relationship between attention and IEDs. Here, we hypothesized that the occurrence of IEDs would be decreased while subjects attend to task-relevant stimuli.

It is also unclear why the electrographic signature of an IED is, at first glance, very brief compared with the duration of the IED’s impact on memory. There is some evidence that the impact of an IED is determined by a slowly varying underlying excitability of the local brain tissue at the moment of the discharge (Chang et al. 2018). It has been proposed that shifts in the excitation/inhibition balance in brain tissue can appear in intracranial electroencephalography (EEG) as a change in the balance of low-frequency and high-frequency activity in the aperiodic signal (Gao et al. 2017). We measured this change using spectral tilt—the slope angle of the log–log power spectrum (de Pesters et al. 2016). Spectral tilt decreases when low-frequency power increases and high-frequency power decreases. We expected that spectral tilt would decrease after an IED, shifting the brain toward a poor memory state of increased low-frequency activity and decreased high-frequency activity (Ezzyat et al. 2017). We hypothesized that IEDs cause transient deregulation of brain activity that persists for longer than the IED, and which can be measured as a change in spectral tilt. Furthermore, we hypothesize that IEDs which cause more disruption of brain activity would cause a greater transient cognitive impairment. If this disruption is the cause of transient cognitive impairment, it would have the most deleterious effects in regions involved in memory and attention. To test this hypothesis, we characterized the impact of IEDs on cognitive performance in unprecedented resolution. Previous studies have been limited in spatial resolution by the relative rarity of IEDs in electrocorticographic recordings, whereas this study uses data from a multicenter collaborative agreement to provide a regional parcellation of IEDs’ impact on memory.

## Materials and Methods

This study was conducted with the approval and supervision of the Dartmouth College Committee for the Protection of Human Subjects. Subjects provided their written informed consent prior to enrollment in this study. Collection of electrophysiological data at multiple institutions as part of the Defense Advanced Research Projects Agency (DARPA) Restoring Active Memory (RAM) collaborative agreement was monitored by the institutional review board of each data collection site’s respective university or hospital (Weidemann et al. 2019).

### Free Recall Task

**Figure 1:**
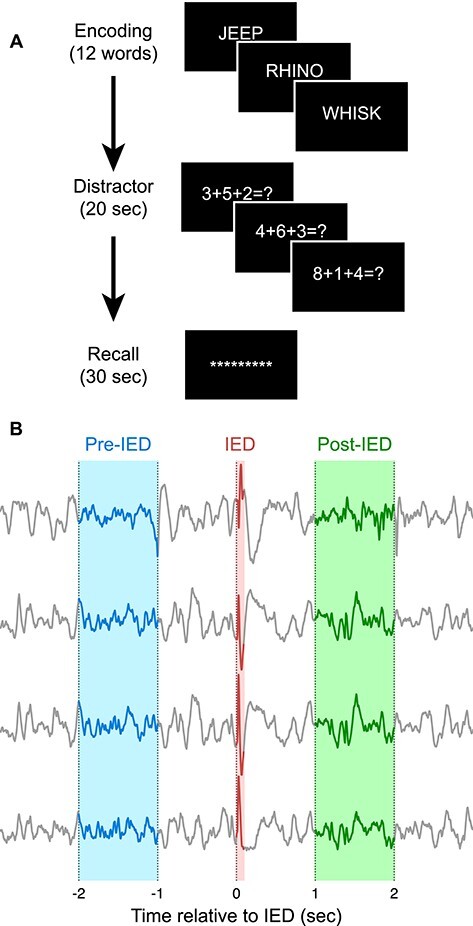
Overview of task and analysis technique. (*A*) Subjects completed a free recall task. In each trial, a series of 12 words were presented on a computer screen. Subjects then completed simple math problems for 20 s as a distractor. Subjects were then prompted to verbally recall words that they remembered from the current trial. (*B*) IEDs were detected within the intracranial EEG recordings, and brain activity surrounding each IED was analyzed. Changes in power were calculated by comparing the period from 2 to 1 s before the IED onset (pre-IED) to the period from 1 to 2 s after the IED onset (post-IED).

Subjects participated in a previously validated free recall task (Ezzyat et al. 2017) using a MacBook Pro with a 15 inch display placed on a tray table. Lists composed of 12 randomly chosen common nouns, which were presented to subjects, with each word appearing for 1600 ms separated by periods of a random duration between 750 and 1000 ms in which the screen is blank (Fig. 1*A*). After 12 words were presented, subjects were presented with simple addition problems for 20 s and typed responses on a keyboard as a distractor. The subjects were then given 30 s to verbally recall any words that they remembered from the previous list. The subjects’ utterances were recorded and manually aligned with the electrophysiological and task data. This procedure was repeated 25 times in each session. In some sessions, sets of 4 words presented in each list were selected from 3 randomly chosen categories to create each list of 12 words, with categories changing between lists (Weidemann et al. 2019).

### Electrophysiological Recordings from Intracranial Monitoring Patients

We conducted our analyses using electrocorticography recordings from 307 subjects with refractory epilepsy who were undergoing intracranial EEG monitoring (Table 1). This dataset was collected as part of the DARPA RAM collaborative agreement at the following epilepsy centers: Columbia University Medical Center, Dartmouth-Hitchcock Medical Center, Emory University Hospital, Hospital of the University of Pennsylvania, Mayo Clinic, National Institutes of Health (NIH), Thomas Jefferson University Hospital, University of Texas Southwestern Medical Center, and University of Washington Medical Center. Recordings were linearly de-trended and notch filtered to remove mains hum at 60 Hz and odd harmonics. All recordings were captured at ≥500 Hz and converted to a common average reference. The recordings were then low-pass filtered using a Butterworth filter at 250 Hz, high-pass filtered using a Butterworth filter at 1 Hz, and downsampled to 500 Hz. Channels with a variance >3 standard deviations distant from the mean channel variance of that recording were excluded.

**Table 1:** Subject demographics

Number of subjects	307
Female	147 (47.9%)
Age distribution	
18–19	7 (2.3%)
20–29	91 (29.6%)
30–39	93 (30.3%)
40–49	69 (22.5%)
50–59	38 (12.4%)
60–69	9 (2.9%)
Age of seizure onset	17.2 ± 12.7
Years of education	13.7 ± 2.3
Left handed	32 (10.4%)
Number of channels	126.1 ± 41.2
Electrode types	
Depth	287 (93.5%)
Strips or grids	109 (35.5%)
Prior resection	47 (15.3%)
Sessions completed	
1	49 (16.0%)
2	71 (23.1%)
3	93 (30.3%)
4	46 (15.0%)
5+	48 (15.6%)
Seizure onset zone	
Focal	61 (19.9%)
Multifocal	93 (30.3%)
Not recorded	153 (49.8%)

### Anatomical Localization of Electrodes

The position of intracranial electrodes was determined using computed tomography (CT)/magnetic resonance co-registration provided with the RAM dataset. *T*_1_-weighted structural preimplantation magnetic resonance imaging scans of each subject were aligned with postimplantation CT scans. Freesurfer was used to calculate hippocampal subfield localization and cortical parcellation. The Desikan–Killiany atlas was used for cortical parcellation and for sorting regions into lobes (Desikan et al. 2006). Final electrode position labels were reviewed by 2 neuroradiologists.

The distribution of recording electrodes was most concentrated in the temporal lobes, but across the set of subjects, there was coverage of most of the brain surface and mesial temporal regions (Supplementary Fig. S1).

### Automated Detection of IEDs

A validated template-based IED detector, described previously (Horak et al. 2017), was applied to generate an initial set of candidate IEDs for each channel. IED candidates from each channel were grouped into a single IED event if they overlapped in time, that is, the IED begins in one channel, and before the end of the initial detection, it is detected in another channel. Artifacts from this set of candidate IEDs were rejected with the following criteria: (a) detections in <4 channels or >45 channels, (b) detections with a duration <10 or >100 ms, (c) detections that exceed the dynamic range of the recording system, and (d) detections that have the same signal value in any channel for >10 samples. The range of channels used for artifact rejection was determined empirically: IED candidates appearing in <4 channels or >45 channels were generally artifactual. Candidate IEDs are filtered by duration during the template matching step; however, a 10–100 ms limit was included to further prevent the inclusion of polyspikes and short duration artifacts. IEDs that occurred within 3 s of another IED were removed to avoid including polyspikes and to prevent contamination of pre-IED and post-IED power spectral analyses.

### Power Spectral Analyses

Frequency transformations of electrocorticography data were computed using the PySpectrum (Cokelaer and Hasch 2017) implementation of a multitaper spectral estimation, using 4 discrete prolate spheroidal sequence windows and 30 logarithmically spaced frequencies from 2 to 120 Hz. The use of multitaper spectral estimation instead of a traditional Fourier transform reduces variance and bias that arises from the boundaries of the finite length electrocorticography (ECoG) signal by averaging several spectral estimates computed using a set of orthogonal window functions. Customary frequency bands were defined as follows: Delta 2–4 Hz, Theta 4–7 Hz, Alpha 8–12 Hz, Beta 12–30 Hz, Gamma 25–40 Hz, High Gamma 40–100 Hz, Ripple 80–120 Hz (Vaz et al. 2019).

### Spectral Tilt

We calculated the spectral tilt of electrocorticography data (Herweg et al. 2020). First, a power spectrum was calculated as described above. The frequency scale and the values of the power spectrum were then log transformed. As the power spectrum of electrocorticography data generally approximates a hyperbolic distribution, this transformation linearized the spectrum. A line was fit to the spectrum using the SciPy least squares linear regression. The inverse tangent of the slope of this line was reported as the spectral tilt for that channel.

### Statistical Modeling and Significance Testing

We used a Poisson generalized linear mixed model with a logarithmic link function to test whether there was a difference in the rate of occurrence of IEDs while a word is presented and during the gaps between word presentation. We modeled the number of discharges that occurred during a single word presentation or gap as a Poisson distributed outcome variable. Epoch (word/gap) was included as a regressor. Subjects were used as a group variable with random intercepts. Since the duration of the word presentation and gaps varied, the duration in seconds was included as an offset, ultimately becoming a multiplicative factor to the expected number of discharges in a trial when passed through the link function. Data from the last word presented in each list was excluded to remove the effects of an audible tone played to signify the end of the recall period in some sessions.

We used a binomial generalized linear mixed model with a logistic link function to test whether the number of IEDs in each region during encoding affected the probability that a word would be recalled. Subjects were used as a group variable with random intercepts. Regions were modeled as random effects with random slopes and intercepts.

We used a linear mixed model to test whether there was a change in band power after an IED. Band power was the outcome variable. Epoch (pre- or post-IED) and brain region (with a nested term for laterality) were modeled as fixed effects with random slopes and intercepts. The differences between subjects were modeled as a random effect applied to all regressor terms.

We used a generalized linear mixed model to test whether changes in band power after an IED are correlated with memory performance. Memory performance was modeled as a Bernoulli predictor variable to encode whether a presented word was recalled or not recalled. Proportion change in band power was normalized using the SciPy implementation of the Box–Cox transform (Virtanen et al. 2020) and included as a fixed effect. Subjects were used as a group variable, with subject–region and subject–laterality interactions as effects with random intercepts.

Linear mixed models were fit using the Python statsmodels package (Seabold and Perktold 2010). Generalized linear mixed models were fit using the R LME4 package (Bates et al. 2015). IEDs in which the proportion change in band power was >3 standard deviations distant from the mean proportion change in any band were excluded. When an IED was detected in multiple electrodes within a single region, the mean proportion change in band power for the electrodes in that region was used in the model. There were between 226 and 8613 IEDs detected in each region.

Statistical significance values appearing in the text and figures were corrected for false discovery rate using the Benjamini–Hochberg technique (Benjamini and Hochberg 1995), unless otherwise stated, using the statsmodels implementation. Statistical significance is indicated using stars as follows: ^*^P < 0.05; ^*^^*^P < 0.01; ^*^^*^^*^P < 0.001.

### Comparisons between Pre-IED and Non-IED Band Power

For each session, the ECoG was processed as described above. Spectrograms were calculated from the *Z*-scored ECoG. Epochs within the session were located containing an 8750 ms stretch of ECoG without IEDs. The temporal mean of the center 750 ms of each epoch’s spectrogram was taken to calculate the band powers for that epoch. Likewise, epochs were computed for a 750 ms window starting at each IED that had a 4000 ms window in both directions without any other IEDs. These sets of band power measurements were compared using a linear mixed model, grouped by subject, session, and channel.

## Results

We used a previously validated automated spike detector (Horak et al. 2017) to identify IEDs in ECoG data from subjects performing a memory task. We began by analyzing the rate of occurrence of IEDs while there is a word stimulus presented on screen compared with blank periods between words to determine whether activation of memory and language regions would decrease IED occurrence in those regions. Next, we examined changes in band power before and after IEDs for evidence of altered brain activity. We then analyzed the impact of IEDs on memory by region, with the hypothesis that there would be a decrease in memory performance when an IED occurs in a region involved in memory. Finally, we examined whether the degree of change in spectral tilt after an IED is predictive of that IED’s impact on memory performance.

### IED Occurrence Decreases during Word Presentation

**Figure 2:**
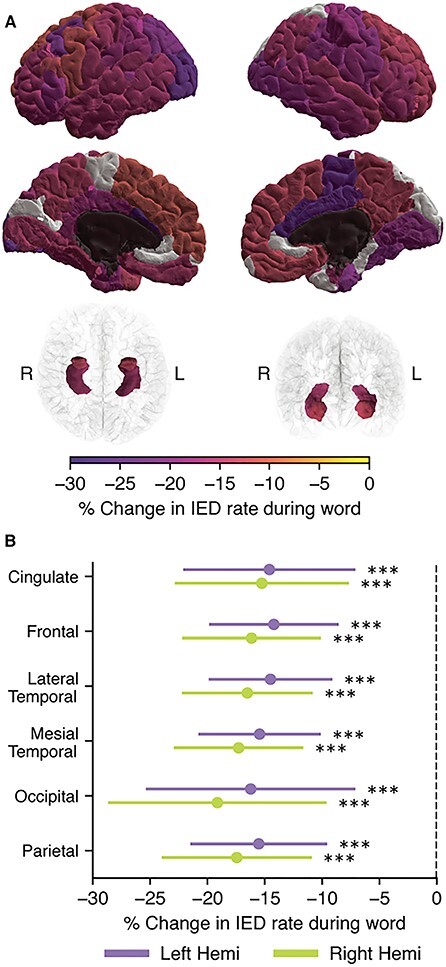
IED occurrence decreases during word presentation compared with periods when the screen is blank. During the encoding phase of the free recall task, subjects were presented with a series of words to remember, with each word separated by a brief gap in which the screen was blank. (*A*) The occurrence of IEDs was analyzed by cortical region during the gap and word periods. Regions in which we recorded from fewer than 15 subjects are shown in dark gray. Areas that were analyzed but had no significant change in IED rate are shown in white. All other regions are significant at *P* < 0.05. More intense colors indicate a larger effect size. (*B*) We also grouped regions by lobe. IED occurred less often in all lobes while a word was onscreen.

A previous study of people with epilepsy found that during a driving task, the occurrence of IEDs was decreased compared with baseline, likely due to attentional effects (Kasteleijn-Nolst Trenité et al. 1987). We hypothesized that activation of brain regions responsible for memory and language processing would transiently decrease the occurrence of IEDs in those regions. To test this, we examined the occurrence of IEDs in different brain regions during the encoding phase of the free recall task when words were presented onscreen compared with when the screen was blank. We found that the rate at which IEDs occurred decreased significantly in most brain regions (Fig. 2) when a word was presented onscreen during the encoding phase of the task compared when the screen was blank. The IED occurrence in the right posterior cingulate was strongly decreased during word presentation relative to other regions (18.5% decrease, *P* < 0.001), as was the occurrence in both the left and right caudal anterior cingulate (21.7% and 19.9% decreases, respectively, *P* < 0.01). There were no regions in which there was a significant increase in IED occurrence during word presentation compared with blank periods.

We also compared the IED rate during words to the IED rate during the distractor and recall phases of the task (Supplementary Fig. S2). IEDs occurred at a significantly lower rate during the distractor and recall phases compared with word presentation. This may be due to the longer continuous duration of the distractor and recall periods amplifying an effect of attention on IED rate.

We sought to determine whether changes in the baseline brain activity could explain the increase in IED rate during gaps. Clips of ECoG were collected from words and gaps that were distant from IEDs and computed the band powers in each clip. When compared, we found that there were significant but small (<5%) decreases in the Alpha, Beta, and Gamma bands during the word epochs compared with gap epochs (Supplementary Fig. S3).

### Brain Activity is Transiently Altered Following an IED

We hypothesized that IEDs would be associated with a lasting increase in low-frequency power and a decrease in spectral tilt, reflecting increased local neuronal synchronization and a decreased ability for tissue to carry out computation effectively. To test this hypothesis, we conducted an analysis of the changes in band power associated with an IED by comparing the power in the 2–1 s before an IED to the power in the 1–2 s after the IED (Fig. 1*B*). The 1 s gap between the IED and the analysis periods prevents a post-IED slow wave, if present, from influencing the power computations. The 1 s duration of the analysis window was chosen as a balance between being long enough to accurately measure band power for the Delta band with reasonable frequency resolution and short enough to meaningfully measure what we expected to be a nonstationary increase in power after an IED.

**Figure 3:**
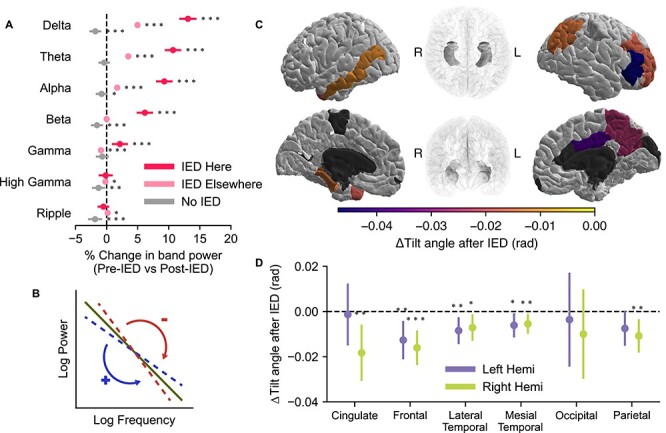
IEDs are associated with increased low-frequency band power and changes in spectral tilt. (*A*) The increase in low-frequency power is more pronounced in channels in which the IED was detected; 95% CI bounds are shown for channels in which an IED was detected, channels in which an IED was not detected but occurred elsewhere, and randomly selected segments of data from time periods in which IEDs were not detected anywhere. (*B*) Spectral tilt is calculated by computing the slope of the power spectrum on a log–log scale. A decrease in spectral tilt corresponds with an increase in low-frequency power compared with high-frequency power. An increase corresponds to a decrease in low-frequency power compared with high-frequency power. (*C*) After an IED, spectral tilt decreases in several brain regions when the IED is detected in that region. Dark gray areas were not analyzed due to insufficient data. Regions that were analyzed but did not have a significant (*P* < 0.05) change are shown in white. (*D*) We additionally analyzed spectral tilt by lobe to boost statistical power. There was a significant decrease in spectral tilt in every lobe except the occipital lobe and the left cingulate and parietal lobes. Due to the increase in statistical power gained by grouping together several regions, spectral tilt changes significantly at the lobe level but not at the region level in some cases.

There was an increase in band power in the Delta, Theta, Alpha, Beta, and Gamma bands, and no change in the High Gamma and Ripple bands (Fig. 3*A*) where the IED was detected. In addition, the magnitude of the increase in band power is largest in the lower frequency bands. The change in band power is larger in electrodes in which the IED was detected than in electrodes in which the IED was not detected.

To ensure that the changes in band power were specific to particular bands, we calculated the coefficient of determination of the IED associated band power changes in each pair of bands (Supplementary Fig. S4). The bands varied largely independently, with a maximum of 45.5% of the variance explained by a neighboring band. Low-frequency bands, which had the largest changes in power with an IED, were particularly mutually independent.

We calculated “spectral tilt,” a measure of the balance of low-frequency oscillations to high-frequency oscillations, for the same periods before and after each IED (Fig. 3*B*). After an IED, there was a significant decrease in the tilt angle, driven by an increase in low-frequency power, in several regions (Fig. 3*C*). Notably, the left middle temporal gyrus, the right pars triangularis, the right precuneus, and the right posterior cingulate gyrus had significantly decreased spectral tilt. There were no regions with a significant increase in spectral tilt. We also grouped the regions by lobe and found that the frontal, mesial temporal, and lateral temporal lobes had decreases in spectral tilt after an IED in both hemispheres (Fig. 3*D*). The cingulate and the parietal lobe only had significant changes in spectral tilt in the right hemisphere, and the occipital lobe did not have significant changes.

**Figure 4:**
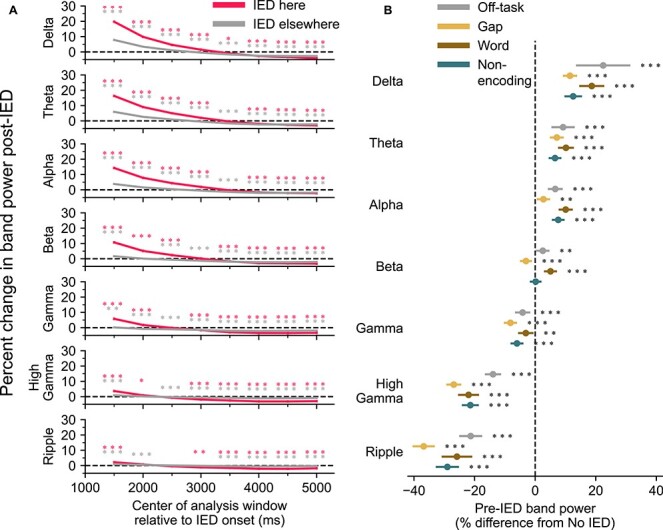
IEDs occur during periods of decreased spectral tilt and are associated with further increases in low-frequency power. (*A*) Changes in post-IED power last several seconds. The pre-IED power was compared against post-IED power computed from a sliding 1 s window. The rise in band power in the lower frequencies decreases over the course of several seconds. (*B*) Pre-IED power was compared with periods of ECoG that were at least 4 s from an IED from the same channel and trial. IEDs occurred during periods of increased low-frequency power and decreased high-frequency power compared with non-IED periods. This finding is consistent across various task epochs and nontask periods.

We investigated the time course of these changes in band power by shifting the 1 s post-IED analysis period farther away from the IED in 500 ms increments (Fig. 4). Low-frequency power remained elevated for several seconds following the IED.

We sought to determine whether the post-IED increase in power observed in Figure 3 could instead reflect a pre-IED decrease in band power. Power spectra from ECoG recordings from each session were calculated, and 750 ms clips (sized to fit within gap epochs) were randomly selected from periods with no IED activity from word presentations, gaps between word presentation, the distractor phase of the task, and the recall phase of the task. Clips were likewise selected from pre-IED epochs from the different phases of the task. Low-frequency power was elevated and high-frequency power suppressed in pre-IED epochs compared with non-IED epochs. This indicates that IEDs tend to occur during periods of decreased spectral tilt. Combined with the results of Figure 3*A*, IEDs occur more often during periods of decreased spectral tilt and are associated with further increases in low-frequency power.

### IED-Associated Changes in Spectral Tilt during Memory Encoding are Not Associated with Modulation of Recall

**Figure 5:**
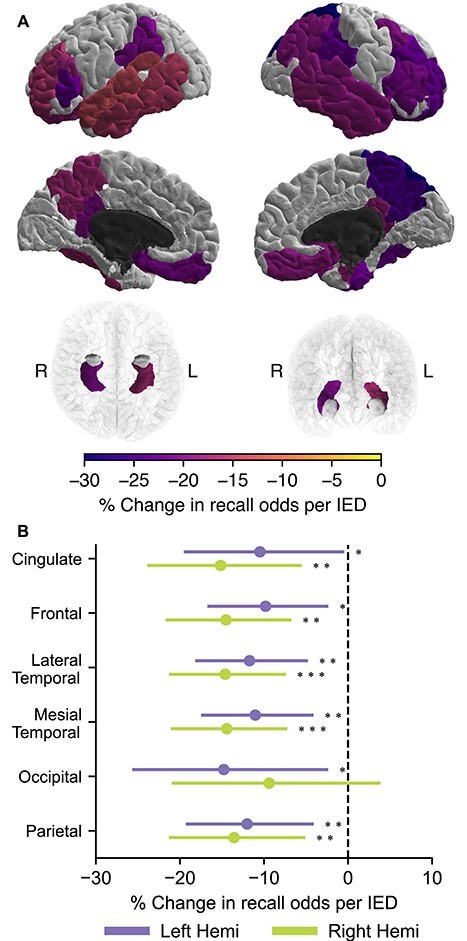
IEDs during memory encoding are associated with decreased recall. (*A*) The probability of recalling a word presented during the encoding phase was modeled with the number of IEDs that occurred as a regressor. IED occurring in several regions throughout the brain, especially in the temporal lobes, significantly decreased the odds of successful recall. Dark gray areas were not analyzed. Regions that were analyzed but did not have a significant (*P* < 0.05) change are shown in white. (*B*) We also grouped the regions by lobe. Compared with (Horak et al. 2017), we found additional areas in the frontal lobe in which IEDs are linked to poor memory performance.

We sought to determine whether the IED-associated changes in band power described above could explain memory deficits common in epilepsy. Toward this end, and as a higher resolution follow-up to our earlier investigation (Horak et al. 2017), we examined the association between the number of IEDs while a word is presented on screen and the probability that the corresponding word is recalled. In contrast to Horak et al., here we had a sufficiently large number of subjects to divide the brain into regions based on the Desikan–Killiany atlas, and to compute separate results for each hemisphere. We expected that the number of IEDs that occurred in memory-related regions while a word was displayed would be inversely related to the likelihood that the subject would recall that word. Like in Horak et al., we found that IEDs occurring during encoding in several mesial temporal lobe and cortical regions were associated with decreased memory performance (Fig. 5*A*), with the largest change in the superior parietal region (23.9% decreased chance of recall). However, in this analysis, we also found that several frontal regions, medial regions, and superior temporal regions were associated with impaired memory. We also grouped the regions into lobes and found that IEDs in each lobe except the occipital lobe were associated with decreased memory performance (Fig. 5*B*). We also found that multiple IEDs during word presentation have a larger impact on memory than single IEDs (Supplementary Fig. S5).

We hypothesized that the impact on memory caused by an IED would be proportional to the IED’s impact on local electrocorticographic activity, measured as changes in band power. We tested this by analyzing IEDs that occurred while a word was being presented to a subject during the encoding phase of the free recall task. For each IED that occurred while a word was being presented to the subject, we calculated the change in band power in the customary bands and stratified the data by whether the word onscreen was successfully recalled by the subject during the recall phase of the trial. We found that there was no significant difference in the band power between IEDs occurring during the presentation of successfully versus unsuccessfully recalled words in any region (Supplementary Fig. S6).

## Discussion

Our hypothesis that IED occurrence would decrease when a word is displayed on screen was supported by the results. However, we did not anticipate that the decrease would be found to varying degree in nearly every region tested. The right posterior cingulate had a particularly strong decrease in IED occurrence during word presentation. This region is part of the default mode network (Raichle et al. 2001). This may suggest that the activity of the default mode network changes during word presentation, possibly as a result of the deployment of attention. Our result differs from an earlier study of an associative memory task (Vivekananda et al. 2019). In contrast to Vivekananda et al., which analyzed the occurrence of IEDs at a trial level, this study analyzed the occurrence of IEDs within and between individual stimuli within trials of a free recall task. Furthermore, we found (Supplementary Fig. S2) that IED occurrence decreases both during the distractor phase of the task (cognitively and visually demanding, not memory dependent) and the recall phase of the task (cognitively but not visually demanding, memory dependent). This suggests that the decrease in IED rate may be due to attention, and not an artifact of visual stimulation or memory. There was also a significant decrease in IEDs during the distractor task, which was less pronounced during the recall task, consistent with a previous finding that IEDs may occur less often during certain visual learning tasks (Aldenkamp et al. 2004).

In this study, we examined IEDs as perturbations of the brain’s activity. We expected that discharges would be followed by a period of hyper-synchronization as the brain attempts to restore normal function, and we observed that IEDs are associated with an increase in band power in low-frequency bands. Although this increase is highest in the electrodes in which the IED was detected, the effects of the IED spread into distant electrodes in which the IED was not detected. This may present a mechanism through which attention decreases the rate of IED occurrence during word presentation. Increased attention, and the corresponding increase in the activity of the ascending reticular activating system, may lead to a shift toward higher frequency cortical activity, that is, a decreased spectral tilt (Moruzzi and Magoun 1949; Englot et al. 2020). The occurrence of IEDs varies with low-frequency activity during non-rapid eye movement (NREM) sleep (Malow et al. 1998; Zubler et al. 2017). IEDs may be more likely to occur during NREM while low-frequency power is increasing (Malow et al. 1997); a result consistent with our finding that pre-IED band power is greater in low frequencies compared with non-IED epochs. Taken together, these results suggest that IEDs occur during periods of decreased spectral tilt and are associated with transient increases in low-frequency power that further decrease spectral tilt.

We found that recall is impaired when an IED occurs in a memory-related region during encoding. This supports previous findings (Horak et al. 2017; Ung et al. 2017) in which IEDs in the temporal and parietal lobes were associated with poor recall. However, the larger sample size of this study revealed that IEDs in much of the frontal lobe can also impair memory. We also found that although memory function is usually lateralized (Kneebone et al. 1995), IEDs in the temporal lobes of both hemispheres impair memory. This may be partially due to selection bias: Subjects whose seizures are thought to come from a temporal lobe with memory function may be more likely to be prescribed intracranial monitoring. However, the robustness of the effect across multiple regions and similarity between hemispheres suggest that there is likely an anatomical basis for this result.

We were surprised to find that the magnitude of the change in spectral tilt after IEDs during encoding did not modulate memory performance. It has been previously shown that IEDs have a nociferous effect on memory (Horak et al. 2017; Kleen and Kirsch 2017), and the other findings of this study also support this conclusion. These findings may suggest that memory formation is extremely sensitive to any disruption, and that an IED of any magnitude at the right time and place is capable of disrupting the delicate process of memory encoding.

The role of IEDs in cognitive impairment and ictogenesis has been the subject of much controversy. It is likely that IEDs have differing effects depending on time-varying excitability of the underlying tissue and the stimuli necessary to move the region closer to or away from criticality (Chang et al. 2018). If the brain does indeed strive for self-organized criticality as theorized by Chang et al., it is possible that the system would exhibit critical slowing down. As the brain becomes more vulnerable to a seizure and farther away from optimal computation, it would presumably take longer to recover from perturbations to its activity.

This study is limited in that there is, as of yet, no plausible means to elicit or suppress IEDs without interfering with brain activity, and therefore this study cannot prove the direction or even the existence of a causal link between IEDs and changes in memory and brain activity. Further research is necessary to determine whether IEDs are the direct cause of transient memory impairment, or whether there is a hidden variable that drives both IEDs and memory impairment. This study is further limited by the selection bias inherent to human intracranial monitoring studies: Electrodes are placed according to a plan that is determined by the subjects’ underlying disease. Further investigation is necessary to determine how these limitations affect the results of this study.

## Code and Data Availability

A representative subset of the data used in this study is available through the DARPA RAM Public Data Release at http://memory. psych.upenn.edu/RAM. Computer code used in this study may be made available upon reasonable request to the corresponding author.

## Significance Statement

Interictal epileptiform discharges (IEDs) occur sporadically in electroencephalography recordings of people with epilepsy. Although the factors that control when IEDs occur are unknown, we found that they are less likely to occur when a subject attends to a stimulus presented on a computer monitor compared with when the monitor is blank. This change is strongest in regions thought to be involved in attention and the default mode network. We found that IEDs are associated with increased low-frequency oscillatory power for long durations relative to the duration of the IED. We also produced a map of regions in which IEDs are associated with memory deficits. Together, these findings suggest a relationship between attention and IEDs.

## Supplementary Material

supplementary_material_tgab019

## Notes

The authors would like to thank the patients and the hospital staff that supported them, without whom this research would be impossible. The views, opinions, and/or findings contained in this material are those of the authors and should not be interpreted as representing the official views or policies of the Department of Defense or the US Government. *Conflict of Interest*: B.C.J. has received research funding from NIH, National Science Foundation, DARPA, Centers for Disease Control and Prevention, Neuropace, Inc., Harvard-Pilgrim, Inc., and the Diamond Foundation. She serves as Associate Editor for the journal Neurology. She has received travel support for activities in her roles as Committee Chair and Vice Chair of the Council of Education of the American Epilepsy Society.
