# Supplementary material for: Interictal Epileptiform Discharges are Task Dependent and are Associated with Lasting Electrocorticographic Changes
Source: Cereb Cortex Commun. 2021 Mar 20;2(2):tgab019. doi: 10.1093/texcom/tgab019 (PMC8152941; doi:10.1093/texcom/tgab019)
Supplement: supplementary_material_tgab019 [file supplementary_material_tgab019.zip › supplementary_material_tgab019.pdf]

Supplementary Material

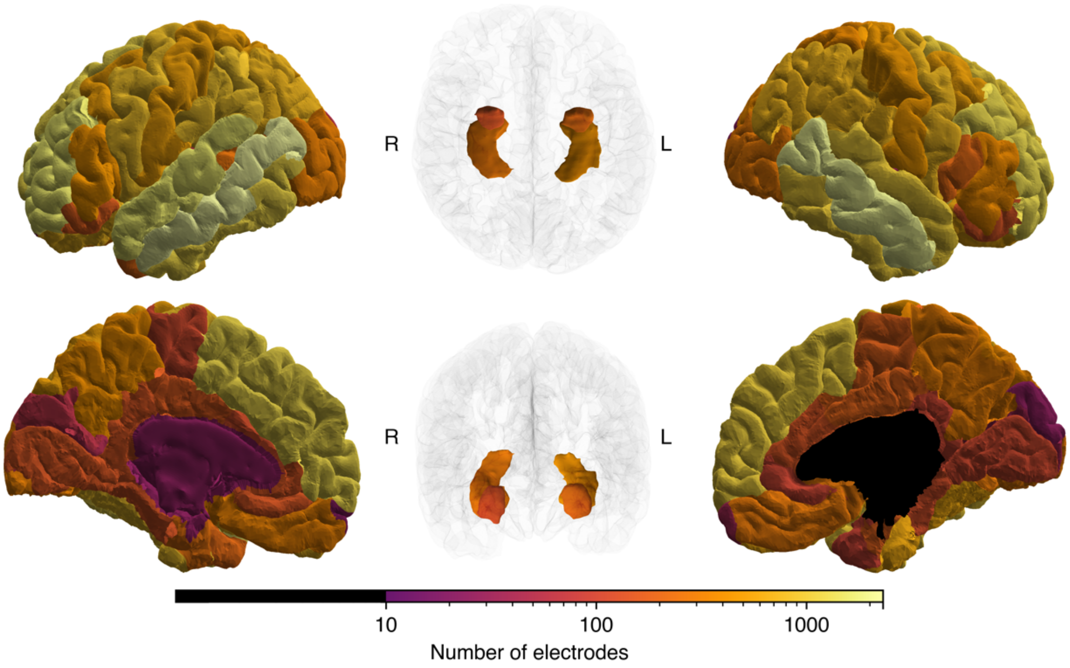

Supplementary Figure 1: Recording electrodes were widely distributed across the brain, with most regions having data from >10 subjects. Recordings were most common in the temporal lobes. Areas with fewer than 10 subjects are shown in black and were excluded from all analyses.

		% Change in IED rate	95% Conf. Int. Lower Bound	95% Conf. Int. Upper Bound	p (B-H Corrected)
Amygdala	L	-9.6	-3.6	-14.6	0.005
	R	-9.6	-2.0	-15.6	0.023
Hippocampus	L	-11.5	-6.8	-15.5	< 0.001
	R	-11.5	-6.4	-15.8	< 0.001
PHC	L	-8.6	1.2	-16.0	0.098
	R	-8.8	2.5	-16.8	0.13
PRC	L	-8.3	-1.6	-13.7	0.024
	R	-12.1	-6.5	-16.7	< 0.001
SUB	L	-11.3	-1.9	-18.3	0.029
	R	-12.0	-5.0	-17.6	0.003
bankssts	L	-13.5	-7.3	-18.5	< 0.001
	R	-13.6	-5.9	-19.5	0.003
caudalanteriorcingulate	L	-21.7	-13.9	-27.4	< 0.001
	R	-19.9	-10.2	-26.6	0.001
caudalmiddlefrontal	L	-15.7	-9.9	-20.3	< 0.001
	R	-10.0	-3.5	-15.3	0.007
cuneus	L	-12.6	1.8	-21.9	0.094
	R	2.5	36.5	-14.7	0.831
entorhinal	L	-13.0	-7.7	-17.3	< 0.001
	R	-11.3	-4.5	-16.8	0.004
frontalpole	L	-13.0	3.3	-23.0	0.119
	R	-9.9	17.6	-23.7	0.401
fusiform	L	-11.7	-7.5	-15.2	< 0.001
	R	-14.7	-10.6	-18.2	< 0.001
inferiorparietal	L	-14.7	-9.8	-18.7	< 0.001
	R	-11.2	-5.5	-15.9	< 0.001
inferiortemporal	L	-11.5	-7.3	-15.2	< 0.001
	R	-13.8	-9.6	-17.4	< 0.001
insula	L	-11.2	-5.9	-15.6	< 0.001
	R	-11.2	-6.1	-15.5	< 0.001
isthmuscingulate	L	-12.3	-5.4	-17.9	0.003
	R	-9.6	-1.9	-15.7	0.024
lateraloccipital	L	-18.0	-9.5	-24.3	< 0.001
	R	-14.2	-6.2	-20.4	0.003
lateralorbitofrontal	L	-12.0	-7.2	-16.1	< 0.001
	R	-10.1	-3.7	-15.3	0.005
lingual	L	-10.8	-2.2	-17.4	0.024
	R	-13.8	-4.5	-20.7	0.01
medialorbitofrontal	L	-10.8	-5.1	-15.5	0.001
	R	-9.2	-2.3	-14.8	0.016
middletemporal	L	-11.4	-7.5	-14.8	< 0.001

	R	-12.2	-8.1	-15.7	< 0.001
paracentral	L	-4.3	15.8	-16.5	0.623
	R	-18.4	-10.3	-24.3	< 0.001
parahippocampal	L	-10.7	-2.9	-16.7	0.014
	R	-8.2	1.6	-15.6	0.11
parsopercularis	L	-9.1	-3.3	-14.0	0.005
	R	-11.4	-4.8	-16.7	0.003
parsorbitalis	L	-12.4	-5.8	-17.7	0.002
	R	-11.9	-2.8	-18.7	0.019
parstriangularis	L	-13.6	-7.8	-18.4	< 0.001
	R	-11.8	-4.6	-17.4	0.005
pericalcarine	L	-12.8	3.3	-22.8	0.119
	R	-19.6	3.7	-31.1	0.098
postcentral	L	-11.1	-5.0	-16.1	0.002
	R	-12.9	-5.9	-18.4	0.002
posteriorcingulate	L	-12.8	-3.1	-20.0	0.019
	R	-18.5	-10.8	-24.3	< 0.001
precentral	L	-8.5	-1.7	-14.1	0.024
	R	-13.7	-7.8	-18.5	< 0.001
precuneus	L	-10.2	-4.3	-15.0	0.003
	R	-9.7	-2.4	-15.4	0.016
rostralanteriorcingulate	L	-5.5	7.1	-14.4	0.374
	R	-5.3	7.5	-14.3	0.395
rostralmiddlefrontal	L	-11.4	-6.2	-15.8	< 0.001
	R	-10.1	-4.6	-14.6	0.001
superiorfrontal	L	-7.7	-0.5	-13.5	0.047
	R	-9.5	-2.4	-15.2	0.016
superiorparietal	L	-11.9	-6.5	-16.4	< 0.001
	R	-7.6	1.3	-14.3	0.104
superiortemporal	L	-11.3	-7.2	-14.8	< 0.001
	R	-10.7	-5.6	-15.0	< 0.001
supramarginal	L	-12.7	-8.0	-16.6	< 0.001
	R	-13.7	-8.3	-18.2	< 0.001
temporalpole	L	-9.8	-1.5	-16.2	0.031
	R	-13.1	0.7	-22.1	0.074
transversetemporal	L	-3.0	11.4	-13.0	0.642

Supplementary Table 1: Changes in IED rate during words (relative to gaps). This data is the same computation as Figure 2a in tabular form.

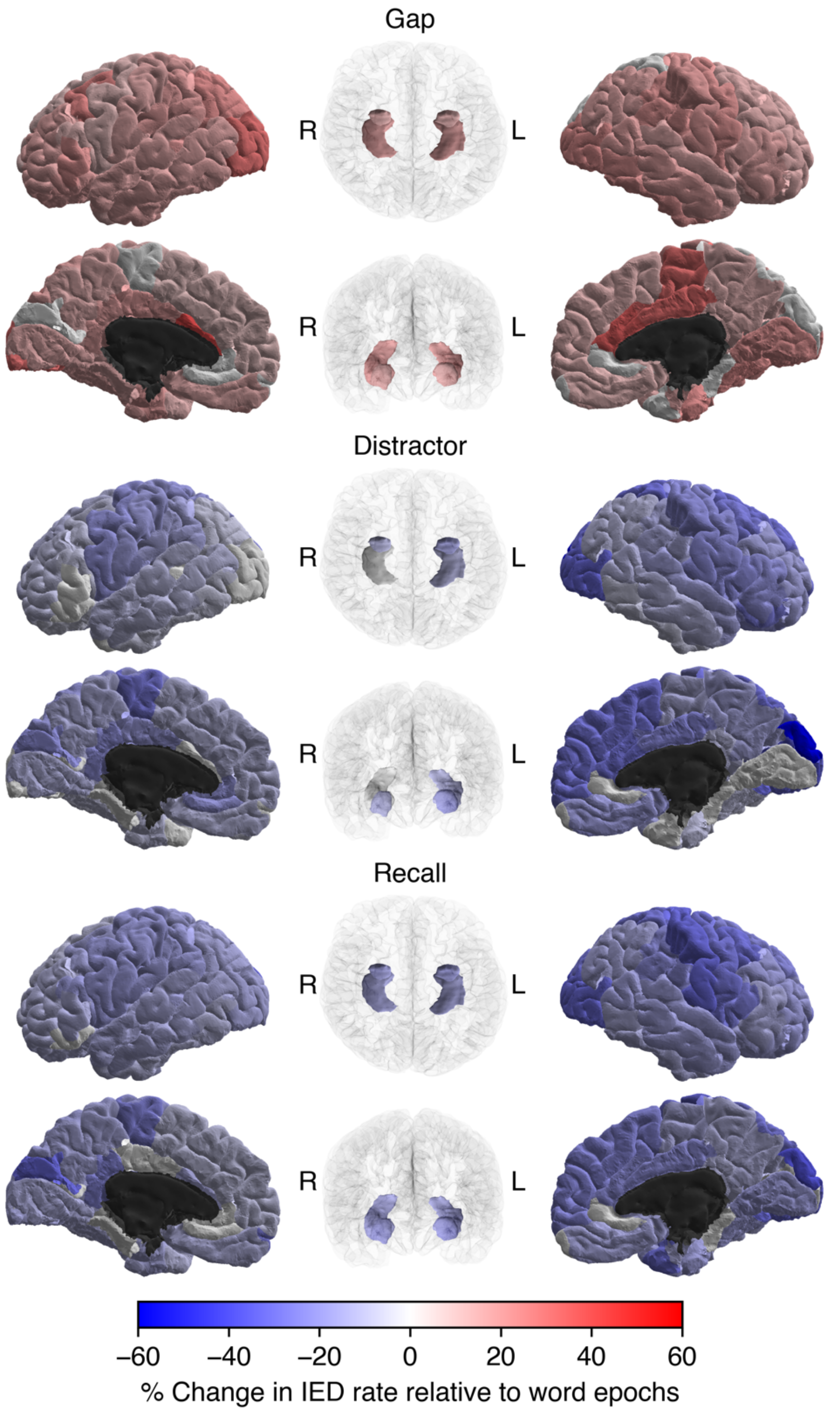

Supplementary Figure 2: Changes in IED rates in experiment epochs relative to word presentation. IEDs are more frequent during gaps than during word presentation, but they are less frequent during the distractor and recall phases of the task.

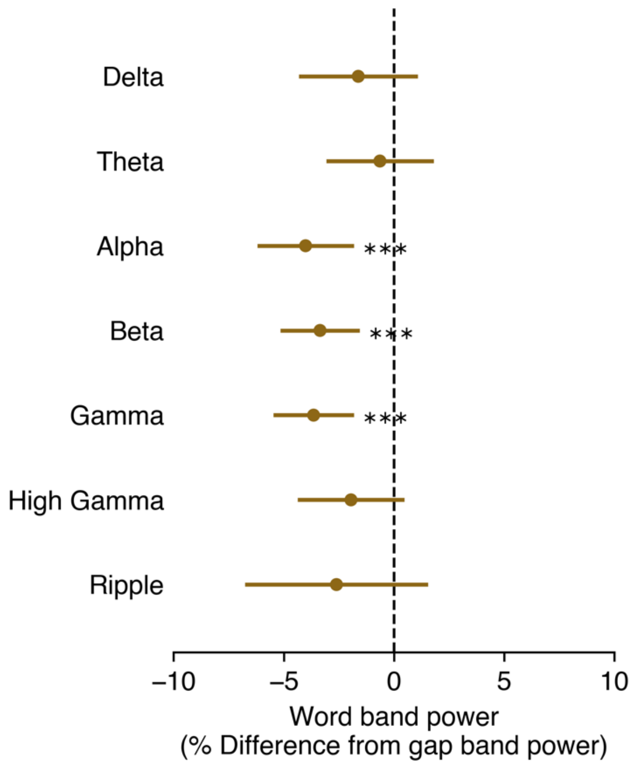

Supplementary Figure 3: Low frequency power is not significantly different during word presentation compared to during gaps between words. A spectrogram was computed using ECoG from each task session, and clips were selected from word presentation and gap epochs. Significance was tested for each band using a linear mixed model grouped by subject and BH corrected. n= ≥137 subjects, ≥782 clips for each group.

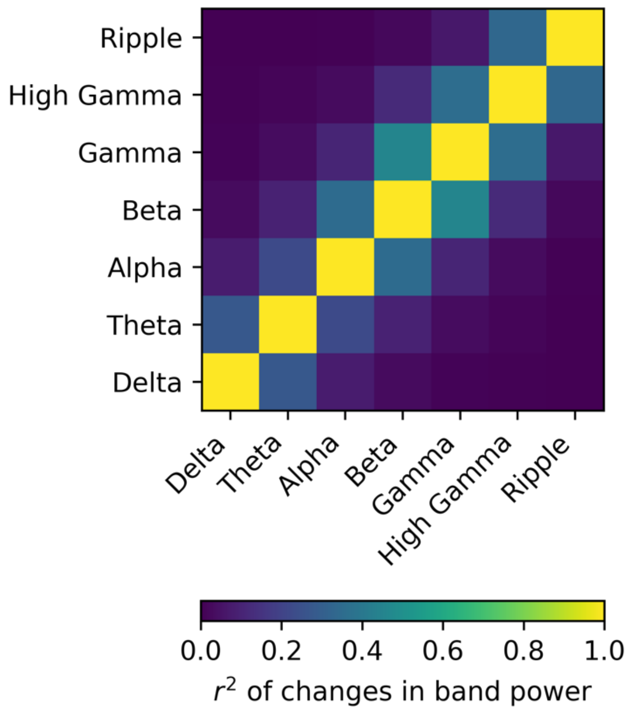

Supplementary Figure 4: Band power changes after an IED are only partially explained by changes in neighboring bands. We computed the change in band power for each channel that recorded an IED, for every IED, and calculated the Pearson correlation between each pair of bands. Neighboring bands explain, at most, 45.5% of the variance in any of the analyzed bands. The lower frequency bands have lower mutual correlation, indicating that they vary independently of each other.

		Percent change in power	95% Conf. Int. Lower Bound	95% Conf. Int. Upper Bound	P (B-H Corrected)
Delta	IED Here	13.1	11.9	14.3	< 0.001
	IED Elsewhere	5.0	4.7	5.2	< 0.001
	No IED	-1.8	-2.6	-1.0	< 0.001
Theta	IED Here	10.7	9.5	11.8	< 0.001
	IED Elsewhere	3.5	3.2	3.7	< 0.001
	No IED	-0.4	-1.1	0.4	0.31
Alpha	IED Here	9.3	8.1	10.5	< 0.001
	IED Elsewhere	1.7	1.5	2.0	< 0.001
	No IED	-0.8	-1.6	0.0	0.05
Beta	IED Here	6.2	5.0	7.3	< 0.001
	IED Elsewhere	0.0	-0.2	0.2	0.961
	No IED	-1.6	-2.3	-0.8	< 0.001
Gamma	IED Here	2.1	1.0	3.3	< 0.001
	IED Elsewhere	-0.9	-1.1	-0.7	< 0.001
	No IED	-0.7	-1.6	0.2	0.131
High Gamma	IED Here	-0.1	-1.1	0.8	0.785
	IED Elsewhere	-0.2	-0.4	0.0	0.045
	No IED	-1.3	-2.2	-0.4	0.006
Ripple	IED Here	-0.5	-1.3	0.3	0.203
	IED Elsewhere	0.2	0.0	0.3	0.025
	No IED	-1.9	-2.8	-0.9	< 0.001

Supplementary Table 2: Changes in band power after an IED. This data is the same computation as Figure 3a in tabular form.

		Change in Tilt Angle (rad)	95% Conf. Int. Lower Bound	95% Conf. Int. Upper Bound	p (B-H Corrected)
Amygdala	L	-0.6	0.1	-1.2	0.15
	R	-0.2	0.5	-0.9	0.713
Hippocampus	L	-0.5	0.1	-1.1	0.223
	R	-0.6	0.0	-1.3	0.144
PHC	L	-1.2	-0.1	-2.3	0.084
	R	-0.9	0.4	-2.2	0.251
PRC	L	-0.5	0.3	-1.4	0.313
	R	-0.4	0.6	-1.4	0.581
SUB	L	0.0	1.3	-1.4	0.974
	R	-0.2	1.0	-1.3	0.855
bankssts	L	-1.1	1.1	-3.4	0.435
	R	0.0	1.7	-1.8	0.975
caudalanteriorcingulate	L	1.4	4.1	-1.3	0.423
caudalmiddlefrontal	L	-1.3	1.0	-3.7	0.388
	R	-1.7	-0.1	-3.3	0.086
cuneus	L	-1.3	1.9	-4.5	0.545
entorhinal	L	-0.6	0.3	-1.4	0.295
	R	-0.1	1.0	-1.2	0.945
fusiform	L	-0.3	1.0	-1.6	0.747
	R	-0.4	0.5	-1.3	0.528
inferiorparietal	L	-1.7	-0.3	-3.1	0.062
	R	-1.4	-0.5	-2.3	0.014
inferiortemporal	L	-0.9	0.2	-1.9	0.182
	R	-1.1	-0.1	-2.1	0.081
insula	L	-0.3	0.6	-1.2	0.615
	R	-1.2	0.0	-2.4	0.118
isthmuscingulate	L	-0.6	2.0	-3.2	0.748
	R	-1.2	0.5	-2.9	0.251
lateraloccipital	L	0.3	3.6	-3.0	0.917
	R	-1.0	2.0	-4.1	0.632
lateralorbitofrontal	L	-1.6	-0.1	-3.1	0.084
	R	-1.7	-0.2	-3.2	0.074
lingual	L	-0.6	1.4	-2.6	0.681
	R	-1.0	1.4	-3.4	0.538
medialorbitofrontal	L	-1.7	0.5	-3.8	0.223
	R	-1.1	0.5	-2.7	0.272
middletemporal	L	-1.1	-0.4	-1.7	0.01
	R	-0.7	-0.1	-1.3	0.069
paracentral	R	0.0	3.6	-3.5	0.985
parahippocampal	L	-1.4	-0.6	-2.1	0.005

	R	-0.6	0.4	-1.5	0.385
parsopercularis	L	-1.2	1.0	-3.4	0.408
	R	-0.6	1.6	-2.7	0.719
parsorbitalis	L	-0.8	2.5	-4.1	0.735
	R	-1.7	0.8	-4.2	0.266
parstriangularis	L	-2.8	1.0	-6.6	0.247
	R	-4.7	-1.5	-7.9	0.016
postcentral	L	-0.1	2.0	-2.3	0.945
	R	-0.9	0.8	-2.7	0.415
posteriorcingulate	L	-0.4	1.3	-2.1	0.735
	R	-3.9	-2.0	-5.8	< 0.001
precentral	L	-0.4	1.4	-2.2	0.775
	R	-1.3	0.0	-2.7	0.13
precuneus	L	-1.0	0.3	-2.3	0.239
	R	-2.5	-0.8	-4.1	0.015
rostralanteriorcingulate	L	1.4	7.7	-4.9	0.762
	R	-0.2	3.2	-3.5	0.964
rostralmiddlefrontal	L	-1.6	-0.1	-3.1	0.086
	R	-1.7	-0.3	-3.0	0.048
superiorfrontal	L	-1.2	0.4	-2.8	0.24
	R	-1.9	0.6	-4.3	0.231
superiorparietal	L	-0.6	0.4	-1.6	0.37
	R	-1.5	0.3	-3.2	0.189
superiortemporal	L	-0.5	0.2	-1.3	0.255
	R	-0.2	1.0	-1.4	0.855
supramarginal	L	-0.4	0.7	-1.6	0.581
	R	-0.1	1.2	-1.4	0.948
temporalpole	L	-1.8	-0.7	-2.9	0.006
	R	-0.2	1.5	-1.9	0.897
transversetemporal	L	-0.7	2.0	-3.3	0.735

Supplementary Table 3: Regional changes in spectral tilt after an IED. This data is the same computation as Figure 3c in tabular form.

		% Change in Recall Probability	95% Conf. Int. Lower Bound	95% Conf. Int. Upper Bound	p (B-H Corrected)
Amygdala	L	-6.0	-16.2	4.2	0.302
	R	-8.7	-21.6	4.2	0.25
Hippocampus	L	-15.3	-23.6	-7.0	0.003
	R	-15.5	-24.5	-6.6	0.004
PHC	L	-11.9	-26.3	2.5	0.165
	R	-14.4	-30.2	1.3	0.123
PRC	L	-24.0	-35.0	-13.0	0.001
	R	-19.7	-29.9	-9.4	0.003
SUB	L	-18.7	-34.6	-2.9	0.045
	R	-16.7	-28.8	-4.6	0.018
bankssts	L	-8.8	-21.1	3.4	0.219
	R	-15.1	-31.1	0.9	0.113
caudalanteriorcingulate	L	9.0	-10.8	28.7	0.429
	R	21.3	-1.7	44.3	0.12
caudalmiddlefrontal	L	0.0	-12.2	12.2	0.999
	R	-17.7	-29.3	-6.0	0.009
cuneus	L	-14.6	-40.9	11.6	0.331
	R	3.2	-27.7	34.1	0.85
entorhinal	L	-10.2	-20.2	-0.1	0.091
	R	-15.4	-28.2	-2.7	0.04
frontalpole	L	11.2	-15.2	37.6	0.461
	R	-39.9	-95.4	15.6	0.219
fusiform	L	-14.1	-22.0	-6.1	0.004
	R	-17.9	-26.6	-9.1	0.002
inferiorparietal	L	-8.4	-18.7	2.0	0.172
	R	-15.6	-26.6	-4.7	0.014
inferiortemporal	L	-12.5	-20.4	-4.5	0.008
	R	-14.9	-23.8	-6.0	0.005
insula	L	-15.1	-24.9	-5.3	0.009
	R	-12.4	-21.9	-2.9	0.025
isthmuscingulate	L	-15.1	-28.8	-1.3	0.065
	R	-14.8	-27.5	-2.0	0.049
lateraloccipital	L	-17.0	-39.8	5.7	0.203
	R	-8.2	-24.5	8.0	0.379
lateralorbitofrontal	L	-16.9	-26.4	-7.4	0.004
	R	-18.1	-29.7	-6.5	0.008
lingual	L	-12.8	-28.4	2.7	0.165
	R	-10.9	-28.7	6.8	0.292
medialorbitofrontal	L	-20.3	-30.7	-9.9	0.003

	R	-15.9	-28.1	-3.8	0.025
middletemporal	L	-13.1	-20.6	-5.5	0.004
	R	-14.9	-22.9	-6.8	0.003
paracentral	L	21.2	-1.2	43.5	0.113
	R	-11.8	-31.5	8.0	0.301
parahippocampal	L	-10.2	-23.6	3.2	0.2
	R	-12.8	-27.1	1.6	0.133
parsopercularis	L	-15.8	-25.6	-6.0	0.007
	R	-18.3	-30.6	-5.9	0.011
parsorbitalis	L	-16.8	-30.1	-3.5	0.03
	R	-11.1	-29.1	7.0	0.292
parstriangularis	L	-21.0	-33.3	-8.7	0.005
	R	-18.8	-32.9	-4.7	0.023
postcentral	L	-4.0	-15.0	7.0	0.525
	R	-6.5	-19.6	6.5	0.382
posteriorcingulate	L	5.0	-12.2	22.1	0.62
	R	-2.2	-21.0	16.6	0.85
precentral	L	-2.5	-13.9	8.9	0.717
	R	-12.1	-24.4	0.3	0.102
precuneus	L	-15.2	-26.0	-4.4	0.015
	R	-21.4	-34.8	-7.9	0.007
rostralanteriorcingulate	L	-2.6	-18.1	13.0	0.788
	R	-19.4	-37.4	-1.3	0.071
rostralmiddlefrontal	L	-14.6	-24.5	-4.8	0.011
	R	-19.3	-29.5	-9.1	0.003
superiorfrontal	L	-6.7	-17.7	4.2	0.292
	R	-8.8	-21.1	3.5	0.219
superiorparietal	L	-8.6	-19.0	1.8	0.165
	R	-26.7	-41.6	-11.8	0.004
superiortemporal	L	-12.2	-20.0	-4.4	0.008
	R	-15.9	-25.5	-6.4	0.005
supramarginal	L	-15.5	-24.9	-6.0	0.006
	R	-20.0	-31.4	-8.7	0.004
temporalpole	L	-14.8	-28.8	-0.7	0.078
	R	-7.9	-28.9	13.1	0.513
transversetemporal	L	-14.9	-33.6	3.9	0.179
	R	4.0	-32.6	40.6	0.85

Supplementary Table 4: Change in recall odds when there is an IED in each region of interest during word presentation. This data is the same computation as Figure 5a in tabular form.

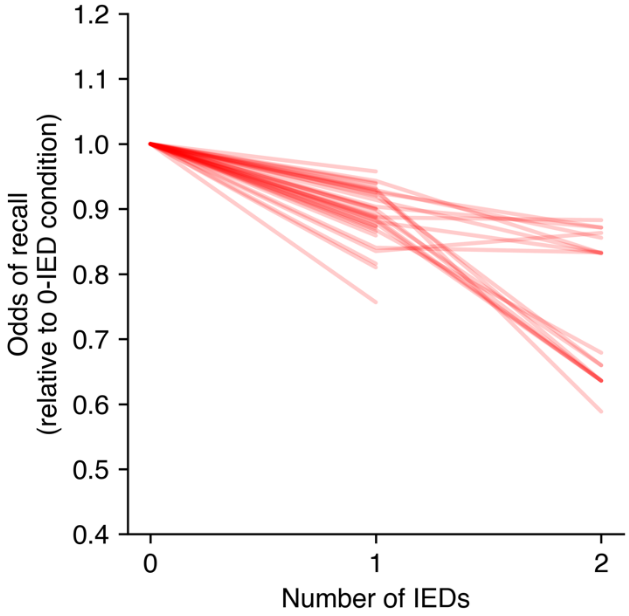

Supplementary Figure 5: Increasing numbers of IEDs during word presentation negatively impact recall. For regions in which IEDs are significantly correlated with memory deficit, we computed the mean recall probability. Regions with fewer than 3 subjects and fewer than 5 word presentations for each number of IEDs were excluded. Recall probability was normalized per region to the zero-IED recall rate for that region. Generally, memory performance when there were two IEDs during a word presentation was worse than when there was only one IED during word presentation.

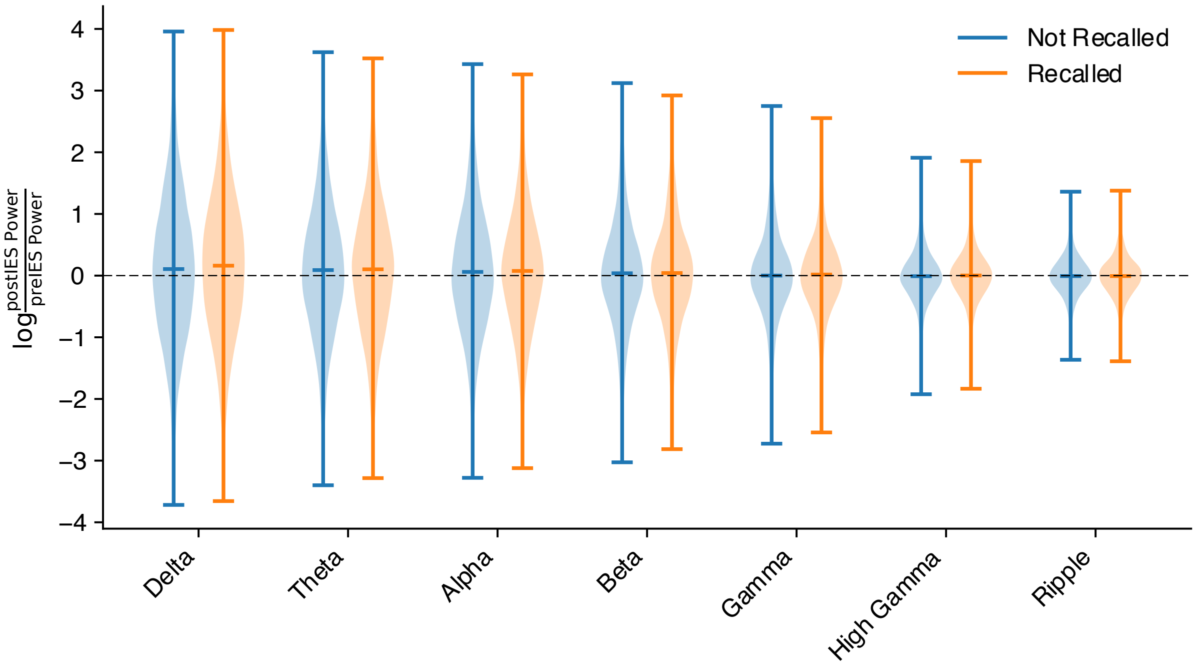

Supplementary Figure 6: When comparing IEDs that occurred during word presentation, the magnitude of band power change associated with the IED is not significantly correlated with the odds of recalling that word. Comparing IED associated change in band power between recalled and not recalled conditions, $p > 0.05$ for all bands.
